# Primary care doctors’ understandings of and strategies to tackle health inequalities: a qualitative study

**DOI:** 10.1017/S146342361800052X

**Published:** 2019-05-21

**Authors:** Mark Exworthy, Victoria Morcillo

**Affiliations:** 1 Health Services Management Centre, University of Birmingham, Birmingham, UK; 2 Faculty of Medicine, Autonomous University of Madrid, Madrid, Spain

**Keywords:** doctors, health inequalities, medical responsibility, primary care

## Abstract

**Aim:**

To examine general practitioners’ knowledge of and their role in tackling health inequalities, in relation to their professional responsibilities.

**Background:**

Primary care is often seen as being in the frontline of addressing health inequalities and the social determinants of health (SDH).

**Methods:**

A qualitative study with a maximum variety sample of English General Practitioners (GPs). In-depth, semi-structured interviews were held with 13 GPs in various geographical settings; they lasted between 30 and 70 min. Interviews were audio-recorded and transcribed. The analysis involved a constant comparison process undertaken by both authors to reveal key themes.

**Findings:**

GPs’ understanding of health inequalities reflected numerous perspectives on the SDH and they employ various different strategies in tackling them. This study revealed that GPs’ strategies were changing the nature of (medical) professionalism in primary care. We locate these findings in relation to Gruen’s model of professional responsibility (comprising a distinction between obligation and aspiration, and between patient advocacy, community participation and political involvement). We conclude that these GPs do not exploit the full potential of their contribution to tackling health inequalities. These findings have implication for policy and practice in other practitioners and in other health systems, as they seek to tackle health inequalities.

This paper examines the ways in which health inequalities are being tackled by primary care doctors (General Practitioners; GPs), through their understandings and actions in tackling those inequalities which they observe in their practice. Although the study draws on GPs in England, we interpret their views and actions in terms of a conceptual framework of professional responsibility. Despite the growing evidence about the causes and manifestations of health inequalities in the United Kingdom and internationally, relatively little attention has been devoted to how inequalities might be remedied and what consequences for professional responsibility might follow.

This paper comprises three sections. The first describes the role of GPs in contributing to and tackling health inequalities. It elaborates a conceptual model to explain where GPs’ responsibilities could and to be located in relation to tackling health inequalities. The second section presents the methods and findings from an empirical study involving in-depth interviews with a diverse sample of GPs. The third section discusses these findings in terms of further initiatives to tackle inequalities and the implications of the conceptual model.

## Health inequalities in general practice

Health inequalities are the systematic and potentially remediable differences in one or more aspects of health across populations or population groups defined socially, economically, demographically, or geographically (Starfield and Birn, 2007; Starfield, 2011). This definition conceptualizes health inequalities in terms of individual factors (age, sex, genetic) within a nested hierarchy of lifestyle factors, social and community factors, and proximal (living and working conditions) and distal social determinants of health (SDH) (socioeconomic, cultural, environmental) (Dahlgren and Whitehead, 1991).

Starfield’s definition points to the potential alleviation of these ‘differences’ by concerted social action (Fisher and Chanan, 2015). Despite the mounting evidence about the causes and manifestations of health inequalities (Wanless, 2004; Marmot *et al*., 2010), the evidence is less strong about how such action might be devised and implemented. For example, the practical barriers to addressing inequalities by health professionals (and others) have often been overlooked (Mercer and Watt, 2007; Popay *et al*., 2007). Therefore, it is important to understand better how clinical professionals interpret health inequalities and act to address them.

As the majority of patient contacts with health services take place in primary care, it can potentially alleviate health inequalities (Starfield, 1998, 2007; World Health Organization (WHO), 2008; Hutt and Gilmour, 2010). For example, primary care can enable access to services for patients with undifferentiated need. However, primary care also plays a role in generating and sustaining such inequalities. The geographical distribution of primary care doctors, for example, is often skewed away from areas of highest need (Shi and Starfield, 2001; Starfield, 2011), often due to resource allocation formulae (Barr *et al*., 2014). Such examples are redolent of the inverse care law (Hart, 1971). Some initiatives, such as those of the Scottish Government, have begun to try to solve this problem (Watt, 2013; Blane *et al*., 2017).

Primary care offers the potential for the holistic treatment of health needs, recognizing the SDH which have given rise to that need (McCarron and Yates, 2000; Public Health England, 2017). A robust infrastructure of primary care has the potential for addressing broader health inequalities (WHO, 2008; Starfield, 2011). Notwithstanding the inverse care law and wider structural issues (Raphael, 2011), primary care has thus often been seen as a suitable n setting from which to tackle health inequalities (Allen *et al*., 2013).

In their daily practice, GPs face the effects of the SDH which have consequences for health inequalities. Patients’ socioeconomic status (SES) affects GPs’ clinical decision-making and can, therefore, influence health inequalities (Eisenberg, 1979; Clark *et al*., 1991; Chard *et al*., 1999). For example, in deprived areas, GPs see patients with more psychosocial problems but generally devote less time per patient consultation (O’Brien *et al*., 2011). In addition, patients may be less willing to raise their issues with the GP as they lack confidence in the GP or the general practice setting (Goodhart *et al*., 1999). Some studies show a negative association between low SES and consultation length, further treatment and on-going care (Hartley *et al*., 1984; Reid *et al*., 1999; Mercer and Watt, 2007). Popay *et al.* (2007) identified factors which might explain such patterns. They included a lack of knowledge among GPs about how to respond to social problems. This made them less likely to probe patients about wider concerns (beyond the reasons for their visit to the GP). They also cited evidence that GPs are more likely to offer reassurance than practical assistance or referral, possibly due to limited knowledge about the local availability of resources. This may be because GPs feel uncomfortable or even incapable in addressing health inequalities (British Medical Association, 2011). It may also reflect a sense of social or cultural distance between the GP and their patients (Kikano *et al*., 1996; Robinson and Phillips, 2003), in terms of differences in income, education and social capital. Such factors interact to generate and sustain health inequalities.

### Explaining action on health inequalities in terms of medical responsibility

A greater appreciation of GPs’ perceptions could have implications for the ways in which GPs act to tackle health inequalities. Their actions to address health inequalities are predicated upon their own understandings of and explanations of the causes and solutions to such inequalities (Mackenzie *et al*., 2017). Specifically, GPs’ perceptions of their responsibilities for tackling health inequalities have remained under-explored. Proposing a conceptual model, Gruen et al. argued that

‘The ways in which socioeconomic factors influence individual patients’ health are shown in expanding domains depicting the proximity of each to physicians’ core responsibility for patient care’ (2004: 95).

**Fig. 1:**
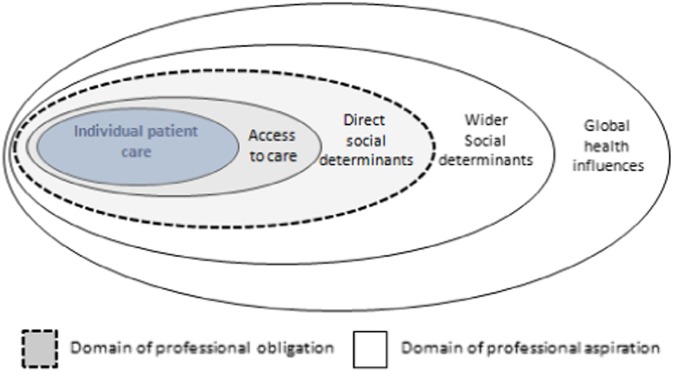
Model of physician responsibility (Gruen *et al*., 2004).

They concluded that doctors’ responsibilities centred on individual patient care, access to that care and direct socioeconomic factors (Figure 1). Doctors’ public roles might also involve ‘advocacy for and participation in improving the aspects of communities that affect the health of individuals’ (p. 94). This is broadly akin to a public health approach (Taylor *et al*., 1998).

**Fig. 2:**
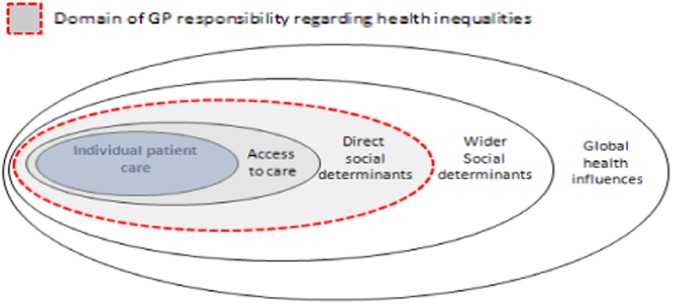
Model of GP responsibility relating to influences on health inequalities Source: Adapted from Gruen *et al.* (2004).

Here, we adapt this conceptual model (Furler *et al*., 2007; Alexander *et al*., 2008) but we examine health inequalities and SDH, rather just simply ‘influences on health’. We retain the concentric, expanding domains as heuristic devices to explain GPs’ justifications for their practices. *A priori*, we begin with the division between obligation and aspiration (Gruen *et al*., 2004). Whilst obligation refers to a normative duty, aspiration implies a more pragmatic decision influenced by personal, organizational and structural factors. Moreover, we also adopt the distinction between patient advocacy, community participation and political involvement (Gruen *et al*., 2006). Here, patient advocacy refers to the ‘needs of individual patients’ (p. 97), participation seeks to improve the aspects of communities that affect the health of individuals and political involvement denotes the actions to shape ‘understandings, beliefs, practice and policies in external institutions’ (p. 97). We accept that the distinction between these categories can be blurred. For example, advocacy might be collective on behalf of a group of patients and political involvement might take place at grassroots level (see Figure 2).

A similar conceptualization is proposed by Raphael (2011) who identifies seven types of discourse of SDH among practitioners and policy-makers. The typology ranges from ‘SDH as identifying those in need of health and social services’ (level 1) to ‘SDH and their distribution result from the power and influence of those who create and benefit from health and social inequalities’ (level 7). Raphael claims that the discourses help in the ‘explication of the SDH concept and their implications for action’. Both conceptualizations address the ways in which practitioners frame their beliefs of and strategies for addressing health inequalities. Whilst Raphael focusses on the legitimation, power and coercion, which underpin health inequalities, Gruen *et al.* address the profession’s underpinning logics of values and interests. However, both can help understand and explain the contribution of practitioners and organizations in tackling the seemingly intractable inequalities and determinants.

## Empirical study of GPs

We established three aims for an empirical investigation: to identify what GPs believe about their own role in (creating, sustaining and ameliorating) health inequalities, to examine their responses and actions to these inequalities and to understand how these responses could be framed in terms of GPs’ responsibility.

### Methodology

To achieve these aims, we devised a qualitative study to examine individuals’ belief, views and actions. We selected a maximum variety sample of GPs who worked in southern England This geographical setting was a pragmatic choice given the location of the researchers but we still sought a diverse sample in terms of location (ie, rural, suburban and inner-city areas) and by deprivation (ie, affluent and deprived neighbourhoods). The sample of GPs was selected on the basis of gender (roughly equal sample of female and male), ethnicity (White, Asian and Black) and seniority (junior, mid-career and senior). The sample was a mix of GP partners (principals) and salaried GPs. The sample was also stratified by organizational size (according to the number of GPs). The GPs we interviewed worked in practices ranging in size from two to six doctors.

Following ethical approval from the lead author’s institution, we undertook two forms of recruitment to this sample. First, we randomly selected practices according to our sample criteria (geography and deprivation). Within each practice, we randomly selected two GPs, on the basis that the practice. We wrote to them and invited them to take part in our study. We received a low response rate which prompted our second strategy which involved a more pragmatic approach of ‘snowballing’ approach by asking participating GPs for suggestions of who else might be interested in taking part. This second strategy generated a better response. We had originally sought a sample of 14–20 GPs but logistical constraints (time of the researchers and their resources) prevented recruitment of a larger sample.

The data collection took place through in-depth, face-to-face, semi-structured interviews with 13 GPs in their practice offices (apart from one, which took place in a communal area) in 2013–2014. We sought permission to audio-record the interviews which took between 30 and 70 min each. Both authors (one of whom is a GP) undertook interviews. These recordings were transcribed by a third party. The following questions were asked to elucidate the study’s aims:

1. How do you define health inequality?
2. From your experience, how do social problems generate health inequalities?
3. Is it sometimes difficult to identify these sorts of problems in practice?
4. What strategies do you use to tackle health inequalities?
5. Do you think these strategies work?
6. Do you think primary care is the best setting to tackle health inequalities?
7. What role should GPs play in tackling health inequalities?

Supplementary questions elaborated GPs’ initial responses. Although the sample was small, we found a degree of saturation in most interview topics after about 10 interviews. Data triangulation (such as observation of patient consultations) might have been possible in a longer, more extensive study.

Analysis was an iterative process, informed by the ‘framework approach’ (Ritchie and Spencer, 1994) comprising familiarization, identifying thematic framework, indexing, charting and interpretation (Gale *et al*., 2013). Each author read transcripts independently. *A priori* themes were compared with themes which emerged from the data. The themes revealed commonalities and differences within and between GPs, and their views and types of strategies. Disagreements in coding and interpretation were discussed by the authors. New interpretations were incorporated in the thematic analysis. Three areas are salient here as they help to shed light on GPs’ beliefs and actions as well as to the application of the Gruen model. They are as follows: GPs’ thinking about inequalities, GPs’ strategies to tackle health inequalities and GPs’ views about wider factors affecting health inequalities. In what follows, we give GPs pseudonyms and summarize their practice setting to contextualize their quotes.

### GPs’ thinking about health inequalities

GPs were asked how they (as individuals) defined health inequalities. The interviewers were not specific about defining health inequalities in their preliminary discussions with GPs in order not to prejudice GPs’ answers. Invariably, GPs spoke about patient care and especially patients’ access to health services, both in primary care and elsewhere. None of them spoke about outcomes in these definitions.

*I suppose to me it would mean that everybody has the same access and receives the same sort of quality and level of treatment if they want it, regardless of, you know, how rich or poor you are, how much English you speak*. (Patrick. Urban, deprived; emphasis added)

*What I see is that some people are more able to access the services they need, they know how to ask, they know how to approach the systems that are there*. (Jacqui. Urban, deprived; emphasis added)

Most (but not all) GPs recognized that health services need to be moderated according to differing levels of need, consistent with the inverse care law (Watt, 2002; Mercer and Watt, 2007) This is also consistent with the notion of proportionate universalism (Marmot *et al*., 2010; Raphael, 2011) although none referred to this term. For example, Elizabeth and Andrew recognized the contrasting needs of their patients. However, Elizabeth adopted a strategy to minimize this, albeit for those already in the waiting room and based on her own subjective perception whereas Andrew’s approach was more passive.

*My worry is those with the loudest voice and the most education tend to be very good at putting their case forward… I try to be very fair on everybody and I try and base my input on who is in greatest need rather than who has the most eloquent voice… In the waiting room, you know, everyone will wait their turn… But I will jump people through the queue if I think they look particularly unwell or need to be seen more quickly.* (Elizabeth. Rural, affluent)

*The generally quite well educated are quite good at getting what they want. That happens in everything, doesn’t it? … So I think there’s an awareness of that but I certainly feel aware of that and that other people are less able to be forceful but when it comes down to the one-to-one, I’m just responding*. (Andrew. Rural, affluent)

None of the interviewees mentioned the social gradient which arguably is a key feature of how health inequalities (and strategies to alleviate them) are currently understood (Graham, 2004; Watt, 2013). This might be important in, for example, comparing responses from Jacqui and Elizabeth (above). Jacqui referred to a lack of social capital of her patients in deprived neighbourhoods, whereas Elizabeth’s patients might reflect a greater capability. In both cases, GPs were focussed on patient advocacy rather.

GPs were asked about their knowledge of health inequalities in general and in their own practice setting. GP discourses which referred to individual causes and solutions would, for example, be more limited than structural ones (Raphael, 2011). So, how did GPs learn about the scale and nature of health need and also inequalities in their practice population? GPs rarely cited research evidence relating to health inequalities to inform themselves; this was surprising. Instead, GPs drew heavily upon tacit knowledge. The length of time as a GP was significant. More junior GPs spoke of the need to learn about and participate in the community they were serving whilst accounts from more senior doctors showed the importance of their experiential knowledge.

‘*I’m sure you’re given theoretical training at medical school [about health inequalities] but I think the practicalities of how that happens is through experience*’. (Zena. Urban, affluent)

‘*I have moved practices as you know, a few times recently and that was always a learning experience - a little bit hard to generalise*’. (Tahir. Urban, deprived)

Likewise, Brian spoke of patients with private health insurance, living in affluent areas. He was asked how he *knew* which patients had insurance.

*So I tend to often suggest but not all the time but often suggest people use their private insurance…. Interviewer: how would you know they had insurance? I would ask… Usually it’s a judgement or if I know their family or if they live somewhere affluent in our population site I might ask them*. (Brian. Urban/suburban, mixed)

He justified this inquiry about patients’ insurance on the basis that it would relieve pressure on the NHS. This strategy is despite speaking just before this quote about his notion of equity: ‘I try to tend to treat everyone the same’.

As another example of GPs’ experience informing their strategy to tackle health inequalities, Jacqui spoke about her strategy to inquire about the social context of one of her patients.

*…that I know a bit more about her now, um, but that was because I asked, I don’t think she’d have told me unless I’d actually sort of taken the initial thing and followed it through and that happens sometimes. You sort of just begin to explore something someone’s dropped in [to the conversation] and if you pursue it, you find it opens up lots of other stuff*. (Jacqui. Urban, deprived; emphasis added)

### GPs’ strategies to tackle health inequalities

GPs’ strategies to tackle health inequalities fell into three main categories: organization of their time, continuity of patient care and integration of services. The exclusive focus on service-related aspects of this theme also prompted questions about the nature and scope of their (professional) responsibility.

GPs described how they used the short consultation times in English general practice (usually 8–10 min/patient) flexibly according to the needs of the patient and to the daily demands. Short consultations tended to reinforce a focus on patient care rather than non-service factors and so a flexible use of time suggested a recognition of the need for different approaches. Zena indicated a further appointment with a patient whose (medical and/or social) needs could not be met within the time of the consultation.

*Some patients if they have clearly got quite a few issues, takes longer. We have 10 minute appointments for patients that clearly have mental health issues… I think it’s just trying to encourage people to come back because within 10 minutes you could run over 20 minutes, 30 minutes… I don’t keep a record of all the patients that I’ve asked to come back*. (Zena. Urban, affluent)

However, this approach is passive as Zena speaks of ‘encouraging’ patients to have follow-up appointments. More active strategies were adopted by Jill and Patrick but still within a focus on patient care within the confines of general practice.

*As a GP, I have tended to make decisions depending on how busy I am, how much I am going to help this person…, if my next patient has turned up or not*. (Jill. Urban, deprived)

*We have a lot of drop in clinics where, you know, you don’t have a set time… but it’s not that there’s a sort of absolute - got to be in and out… And in some senses you have to prioritize because you could - with a lot of this population - spend any amount of time*. (Patrick. Urban, deprived)

These strategies seemed to be highly contingent upon GP availability. Consultation times were linked to the second aspect: continuity of care. Repeat visits (as used by Zena) were used not simply to keep to the clinic schedule but also to build rapport and knowledge of patients’ circumstances. This was helped by continuity of doctor, a practice which is not universal (Mainous *et al*., 2001; Stokes *et al*., 2005).

*Trying to find ways of helping patients to keep coming to the same doctor and we sometimes do it by putting notes saying always book with Dr So-and-so*. (Jacqui. Urban, deprived; emphasis added)

Bec*ause often it’s [general practice] the last bastion… So many people say I’m the only constant. If they go to the hospital, they see a different person, social worker changes every week, carers change, may even like me or hate me, at least, you know, the devil you know better than the devil you don’t!* (Elizabeth. Rural, affluent; emphasis added)

However, whilst the value of repeat visits to the same GP was recognized, this was often hampered by the transitory nature of some patients, especially in urban areas.

Integration of health and social care might also help GPs address the multi-faceted nature of health inequalities (Glasby, 2017). In English general practice, GPs invariably work with practice-based nurses, community nurses (working with new mothers and elderly patients) and social workers. Also, GPs increasingly have links with other public services (such as education or housing departments). However, GPs rarely employ these other staff. In this study, we found that GPs tended to rely mainly on referral to and liaison with other practitioners/agencies. However, among our sample, there appeared to be limited engagement with external agencies about health inequalities or the SDH (in the forms of campaigning against the location of fast food outlets near schools or for lower speed limits near housing, for example). This lack of engagement denotes limited involvement in ‘political’ activities. Whilst Andrew and Brian (among others) were aware of the national political debates on NHS funding, these were more distal factors than, say (local) campaigns and seemed to generate only limited action.

*I think we need to integrate our services and I think that’s really one of the big pluses that might come out of the [NHS] re-organization so I think that could be really good. I think the major obstacle is funding and the [then current health reforms] hopefully will start to move towards… but I think that’s a real big positive that could come out of it*. (Andrew. Rural, affluent)

*I certainly saw an improvement with the previous government in terms of sort of [waiting] times and stuff but there’s a lot more fragmentation of care and so it’s become much more difficult for GPs… And the holistic approach seems to be down to me [he laughs] so I think that’s what happened with the changes*. (Brian. Urban/suburban, mixed)

GPs’ comments on these strategies prompted further questions about how far their role does and should extend. Whilst many were supportive of efforts to tackle health inequalities, workload and administration were cited as why GPs’ further actions were limited. So, what is and should be their professional responsibility with respect to health inequalities? GPs offered different and contradictory answers.

*I suppose yes we have a role; should we have the majority of that role? That I doubt. I would question but yes, we have part of our ethical and our way of practicing as a good doctor is to be aware of the health inequalities and trying to help solve them*. (Elizabeth. Rural, affluent)

*[I] suppose there is a lot that you can do, …I’d love to push for [this] or do something about that, but you know, time is limited. Off the top of my head… over 75% of my time is spent in face-to-face consultations so it doesn’t leave very much for doing- for writing letters and checking the emails - doing all the other bits*. (Kevin. Urban, deprived)

The ramifications of this issue are explored later in the paper.

### Wider institutional factors affecting health inequalities

We were interested in GPs’ views of the wider factors creating and sustaining health inequalities, beyond general practice. For example, GP ‘principals’ often work as independent contractors (rather than NHS employees) but growing numbers are employed as salaried staff. General practice has traditionally been a small business, managing income and expenditure. This institutional status seemed to influence GPs’ views about tackling health inequalities.

*…because I’m a businessman at the same time and my business is the general practice and that’s determined by the number of people that are registered with me or registered with the group and although we’re the one practice in town, it’s an important thing. So I’m reminded, this is a customer as well as a patient. I think that’s important but I hope I have honest conversations with people*. (Andrew. Rural, affluent)

This organizational aspect had consequences for the social/cultural distance between the GP and their patient. For example, Kevin separated his professional and personal life as a ‘coping mechanism’.

*I don’t think I’m cold when it comes to my approach to patients but I’m able to sort of just divorce myself from it once it’s [the working day] over to some extent*. (Kevin. Urban, deprived)

GPs recognized the distance between them and their patients. In terms of SES, for example, Elizabeth cited the pay differential to illustrate this distance.

*I think that the way we’re currently paid and rewarded is very toxic and it’s given the appearance that GPs are very money-grabbing and money orientated and that appearance has some truth to it*. (Elizabeth. Rural, affluent)

Some GPs claimed that organizational changes and policy reforms had not been conducive to better patient care or to tackling health inequalities. GPs in this study were particularly critical of the financial incentive scheme, Quality and Outcomes Framework (QOF) (Doran *et al*., 2011; Dixon *et al*., 2012). QOF is a pay-for-performance scheme which was not specifically designed to reduce inequalities. QOF seemed to affect GPs’ approaches to tackling health inequalities and SDH. However, Gillam *et al.* found that ‘inequalities in processes of care comparing the most and least deprived áreas have narrowed’ (2012: 464).

*…the way I work now is mentally different in that I see people I think who I need to get the QOF points: I need to do this, I need to do that. It’s a massive distraction to what I used to do and what I should be doing which trying to sort things out and trying to do what is right for the patient… A lot of the problems are to do with loneliness and social isolation and housing [but] there’s no QOF point for that… It completely misses the point of that individual and what their real needs are*. (Elizabeth. Rural, affluent)

GPs’ negative comments about QOF outweighed any positive comments about other related reforms such as public health’s new role in local government or the role of Health and Wellbeing Boards, for example. GPs were also acutely aware that many ‘causes of the causes’ lay outside general practice or any local agency. This did not stop efforts by some GPs. However, there was a grudging acceptance that the scope of their work was limited.

‘*Almost every patient that comes into the surgery comes in with some social problem or another and so they come to the GP whether we’re the best person or not to deal with it… There’s not much I can do really that’s the problem. I mean I can refer them, I can send off another letter to somebody else or to the housing or whatever it might be but you know, I’m not really in a good position to deal with that problem but what is does is it takes up a lot of my time*’. (Kevin. Urban, deprived)

Kevin’s conclusion that he was not in a ‘good position’ suggests limits on his responsibilities towards health inequalities and SDH were both personal and structural. By extension, this could apply to the role of general practice more generally.

## Discussion

These findings reveal that GPs’ thinking on health inequalities centred on patient access to care. Views were shaped by the unequal need of patients and GPs’ own experience. GPs’ strategies to tackle inequalities involved adjustment of their consultation time, continuity of care and service integration. Such strategies were shaped by wider structural factors such as their cultural distance from patients and organizational changes and policy reforms.

We did not seek to assess the validity of views nor the efficacy of strategies in tackling health inequalities. Moreover, some GPs may have presented well-rehearsed accounts of their views and strategies (Raphael, 2011). Equally, the study took place in southern England and although there is a higher concentration of more deprived authorities in the north, deprived areas can be found in all regions of England. Corroborative evidence from GPs’ actions (using ethnographic methods rather than relying on interview data) and from a wider sample are warranted. We did not seek to secure empirical generalizability due to the small-scale of the study; rather we attempted to elaborate the theoretical propositions of Gruen *et al*. regarding professional responsibility.

**Fig. 3:**
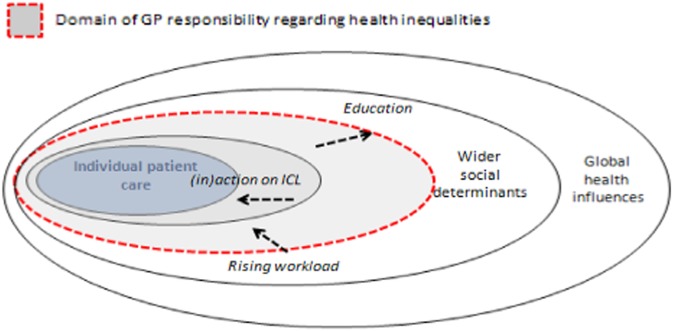
Model of GP responsibility relating to influences on health equalities Source: Adapted from Gruen *et al.* (2004).

These findings reveal GPs’ insights into how such responsibility is formed, evolved and might be shifting. It also highlights areas for further research. The findings confirm a division between obligation and aspiration, and the factors which shape the boundary. The existing boundary identified by Gruen *et al.* (2004) was still relevant but additionally, we note the influence of GPs’ own experience, rising GP workload and the limited action on addressing the inverse care law. These factors affect the position of aspiration/obligation boundary for each GP (Figure 3).

GPs’ experience (including their medical education, continuing professional development and career decisions) shaped their willingness and capability in tackling health inequalities. Rising workload seemed to act as a constraint on GPs’ strategies. GPs were generally confining themselves to individual care and patient access which, in turn, acted as a barrier to tackling the inverse care law. In turn, the individual factors were reinforced by organizational (practice) and structural factors. This echoes Raphael’s (2011) observation of a reluctance to explore the implications of SDH concepts.

These factors modify the model and offer a more dynamic picture of general practice relating to new forms of professionalism (Jones and Green, 2006) and not just in relation to health inequalities and SDH (Raphael, 2011; Peckham *et al*., 2015). The model can also be distilled into three dimensions which cut across the activities of GPs: patient advocacy, community involvement and political participation (Gruen *et al*., 2006). In this sample of English GPs, we found that patient advocacy was a strong theme, whereas community involvement was more limited and political participation was sparse.

Our evidence suggests that a conceptual framework of professional responsibility does equate with our data sufficiently well to merit further investigation.

## Conclusion

This paper demonstrated how English GPs define health inequalities in their daily practice and how this shaped their strategies to tackle them. It concluded that a combination of professional perceptions, organizational reform and changing patient need affected the extent to which GPs felt able to tackle health inequalities. This influenced perceptions of their obligation.

The study generated new understandings about GPs’ roles in tackling health inequalities. Emergent themes were discerned which could, with further research, be applicable elsewhere and in other countries.

In summary, there appeared to be a focus amongst these English GPs into patient advocacy and (limited) community involvement, rather than political participation, with regards to tackling health inequalities.
